# Predictors of Multidrug-Resistant Tuberculosis (MDR-TB) in Sudan

**DOI:** 10.3390/antibiotics8030090

**Published:** 2019-07-09

**Authors:** Monadil H. Ali, Alian A. Alrasheedy, Mohamed Azmi Hassali, Dan Kibuule, Brian Godman

**Affiliations:** 1 Discipline of Social and Administrative Pharmacy, School of Pharmaceutical Sciences, Universiti Sains Malaysia (USM), Minden 11800, Malaysia; 2 Department of Clinical Pharmacy, College of Pharmacy, Northern Border University, 91911 Rafha, Saudi Arabia; 3 Unaizah College of Pharmacy, Qassim University,51911 Qassim, Saudi Arabia; 4 School of Pharmacy, Faculty of Health Sciences, University of Namibia, 340 Mandume Ndemufayo Avenue Pioneers Park, 13301 Windhoek, Namibia; 5 Department of Laboratory Medicine, Division of Clinical Pharmacology, Karolinska Institutet, Karolinska University Hospital Huddinge, SE-141 86 Stockholm, Sweden; 6 Strathclyde Institute of Pharmacy and Biomedical Sciences, University of Strathclyde, Glasgow G4 0RE, UK; 7 School of Pharmacy, Sefako Makgatho Health Sciences University, Ga-Rankuwa, 0204 Pretoria, South Africa

**Keywords:** MDR-TB, Predictors, Sudan, Tuberculosis

## Abstract

Multidrug-resistant tuberculosis (MDR-TB) is a global public health threat and burden on the health system. This is especially the case in high tuberculosis (TB) prevalence countries, such as Sudan. Consequently, this study aimed to ascertain the predictors of MDR-TB in Sudan to provide future guidance. An unmatched case-control study to assess the predictors of MDR-TB infections among the Sudanese population was conducted from August 2017 to January 2018 at Abu-Anga referral hospital. Patients’ data was gathered from patients’ cards and via interviews. A structured pre-validated questionnaire was used to gather pertinent information, which included sociodemographic characteristics and other relevant clinical data. Univariate and multivariate logistic regression analysis was employed to determine the predictors of MDR-TB infection. 76 of the 183 patients interviewed (41.5%) had MDR-TB cases. The independent predictors for MDR-TB were living in rural areas [adjusted odds ratio (aOR) = 3.1 (95% confidence interval (CI): 1.2–8.2)], treatment failure [aOR = 56.9 (10.2–319.2)], and smoking [(aOR = 4 (1.2–13.2)], whereas other sociodemographic factors did not predict MDR-TB. In conclusion, the study showed that a history of smoking, living in rural areas, and a previous treatment failure were the predictors of MDR-TB in Sudan. The latter factors are most likely due to issues that are related to access and adherence to treatment and lifestyle. The existence of any of these factors among newly diagnosed TB patients should alert clinicians for the screening of MDR-TB. The implementation of directly observed treatment (DOT) and health education are crucial in stopping the spread of MDR-TB in Sudan.

## 1. Introduction

The identification and management of tuberculosis (TB) is still a priority area across countries, as it is the second leading cause of death worldwide from infectious diseases, including patients with concomitant HIV and TB [1,2,3,4]. TB is a concern in Sudan, as it is a high TB burden country in the Eastern Mediterranean Region/World Health Organization (EMR/WHO) [5,6]. In 2017, 21054 cases of TB were notified in Sudan [4]. Internal displacement enhances this high incidence of TB in Sudan [7].

Cases of multidrug-resistant tuberculosis (MDR-TB) are rising, which is a growing concern across countries [4,8,9,10,11]. Although access to drug sensitivity tests (DST) for TB has increased, significant gaps remain in the area of diagnosis and management of MDR-TB across countries [12]. In addition, studies in Zimbabwe, South Africa, and Ethiopia associate high treatment default rates with leading to the development of MDR-TB due to the extended treatment period and unwanted side-effects of second-line anti-TB medicines [13,14,15]. Failure to implement Directly Observed Treatment (DOT) also plays a major role in the existence of disease resistance, according to the WHO [16]. Moreover, resistance to anti-TB medicines is a reflection of the poor quality of TB programs in countries and directly impacted by sub-optimal therapeutic practices [17].

The WHO has reported that, between 2000 and 2004, 20% and 2% of TB cases were identified as being resistant to first and second-line medications, respectively [18]. The prevalence of MDR-TB in Sudan is higher among retreated TB cases when compared to the new cases. An epidemiologic study that was conducted in three primary TB management units in Khartoum reported 5% and 24% resistance among new and previously treated cases, respectively [19]_._ In addition, the prevalence of MDR-TB was noted to be 19% among previously treated TB patients and 1.8% among new cases, according to the 2012 Sudan National TB program (NTP) report [20]. However, higher rates were seen in a study that was carried out in north-eastern Sudan at 6% among new cases [21]. This is a concern to both the authorities and patients as the control and treatment of MDR-TB is difficult and costly [22,23,24].

Despite these concerns, to date, the prevalence of MDR-TB in Sudan has not been systematically determined, which is partly due to the limited laboratory capacity [25], although the NTP published its report in 2012. This is a key issue to address as the detection of MDR-TB and its potential predisposing factors helps in informing the instigation of pertinent strategies to control the development of resistance, optimize treatment costs, and lessen the burden of adverse drug reactions that are associated with the management of MDR-TB [22,23,24,26]. Currently, there is only one reference laboratory in Khartoum state that performs cultures and DST for MDR-TB patients. At a minimum, three to six weeks are needed for the notification of drug resistant TB phenotypes by direct and indirect methods, respectively [7]. This calls for systems, such as early notification and prompt management of MDR-TB patients, to counter the spread of the MDR-TB [12,22]. Consequently, the aim of this study was to identify the risk factors that are associated with MDR-TB infections in Sudan, in order to help with future management strategies and to provide guidance on the potential ways to control the spread of MDR-TB in Sudan.

## 2. Methods

### 2.1. Study Setting

This study was conducted at Abu-Anga regional referral hospital, which is located in Omdurman city, Khartoum state, and is the main TB specialized hospital in Sudan. All of the suspected resistant TB cases are referred to this hospital for confirmation, and it is currently the only source for MDR-TB statistics in Sudan. The National TB Reference lab (NTBRL) only processes DST requests from this hospital and the country office of MDR-TB focal person in Sudan is located in this hospital. NTBRL is under the administrative umbrella of the Federal Ministry of Health (FMOH) of Sudan. Sixty-one and thirty-one MDR-TB cases were notified and commenced treatment in the hospital in 2016 and 2017, respectively. Of these 92 cases, 76 agreed to participate in the study, giving a participation rate of 82.6%.

### 2.2. Study Design and Data Collection

An unmatched prospective case-control study was conducted over a six-month period, from August 2017 to January 2018. We adopted this design as it was seen as the most feasible and applicable method for our study. In fact, matching was not possible, as this method assumes a large patient population to select suitable cases for matching or a long period of time for small populations. However, in our study, we have taken measures to reduce the possibility of confounding by recruiting TB retreatment patients as the control. Moreover, multivariate logistic regression diminishes the effect of confounding. A standardized data collection form with the main determinants of MDR-TB being taken from the literature was used to collect data from patients and TB cards. The main outcome variable was a diagnosis of MDR-TB versus non-MDR-TB, i.e., smear-positive retreatment cases. The cases were bacteriologically confirmed to be resistant to at least rifampicin and isoniazid, whereas the controls were category II cases. According to the WHO definition, category II includes previously treated patients (treatment failure, relapse, and defaulters) [27]. The covariates were sociodemographic (e.g., age, sex, family size, occupation, and residence), clinical, and behavioral factors, such as history of imprisonment, drug addiction, smoking, chronic use of antacids, previous TB treatment outcome, history of contact, human immunodeficiency virus/ acquired immunodeficiency syndrome (HIV/AIDS) co-infection, and alcohol. The inclusion of these covariates of MDR-TB in our study is based on numerous globally published studies. The findings from a recent study that was undertaken in East Shoa, Ethiopia, indicated that previous treatment, contact with a known TB patient, rural residency, occupation, and alcohol consumption were all reported as the MDR-TB predisposing factors [28]. Moreover, several studies referred at various levels to the correlation of MDR-TB with certain sociodemographic and clinical factors. These factors included smoking, alcohol, HIV/AIDS, level of education, occupation, previous exposure to TB, and history of TB contact [29,30,31,32,33,34]. In addition, other factors that are related to cultural behaviors in Sudan, and some neighboring countries, such as Ethiopia, including snuff chewing, were included.

The patients were interviewed during their regular visits to the hospital after obtaining both verbal and written consent. A trained medical assistant working at the TB hospital conducted the interviews and kept records. In this study, all of the MDR-TB patients who were prescribed second line anti-TB medicines (i.e., the cases) and all of the patients on category II first-line medicines (i.e., the controls) were targeted. New TB patients were excluded to ensure similarities between the two groups, cases and controls, except for the determinants under investigation. We are also aware that the rate of MDR-TB is much higher among those patients who have been previously exposed to anti-TB medicines and whose treatment outcomes were recoded as poor treatment, in particular, treatment failures [30,34]. The aim was to keep confounding bias to a minimum.

Patients who agreed to participate were recruited. Seventy-six MDR-TB—the cases—and one hundred and seven smear-positive—the controls—were included in the study. Ultimately, a total of 183 participants were recruited in the study.

### 2.3. Statistical Analysis

The data were entered onto IBM-SPSS version 24. Univariate statistical analysis of the association between independent variables, factors under investigation and the dependent variable, and the outcome, were undertaken and the values were considered to be significant at a p-value of less than 5%. Statistical tests, including Chi-square or Fisher’s exact test, were used in the study. Binary logistic regression was also used. Multiple logistic regression analysis was also employed, and the predictors of MDR-TB were identified. The control category (i.e., the retreatment cases) was treated as the reference variable in the outcome section. The MDR-TB status in the outcome variable was coded as one, whereas the retreatment cases were entered as zero. Only the independent variables concluded as being significantly associated in the binary regression analysis were employed in the multiple logistic regression to eliminate the effect of confounding bias. Ultimately, the determinants of MDR-TB development were identified.

### 2.4. Ethical Approval

Ethical approval was granted by the research directorate, Federal Ministry of Health, Sudan, with the approval number: fmoh/nhrc/rd/ec dated 29/07/2017. The purpose and expected outcomes of the study were clearly explained to the participants. Furthermore, the interviewer informed the participants that his/her information would be kept confidential and would not be used for any other purpose. The form includes a briefing about the study, name, and address of the participant, as well as the signatures of participants and the data collector. Only those who agreed to participate and signed the consent form were included in the study.

## 3. Results

### 3.1. Characteristics of the Study Participants

76 (41.5%) of the 183 TB patients who participated were MDR-TB cases when compared to 107 (58.5%) controls, i.e., smear positive retreatment TB patients. 63.9% were aged 18–39 years, and 76.5% were male (Table 1). There were no significant (*p* > 0.05) differences in the baseline socio-demographic and clinical characteristics between the cases and controls, except for residency, history of imprisonment, TB contact, previous treatment outcome, HIV coinfection, alcohol intake, smoking, and asthma (Table 1).

### 3.2. Association of Socioeconomic Factors, Clinical and Behavioral Factors with MDR-TB

On binary logistic regression analysis, the crude odds ratio (OR) showed that smoking (OR 1.93, 95% confidence interval (CI) 1.1–3.5), living in rural areas (OR 4.0, 95% CI 2.1–7.5), and imprisonment (OR 2.3 95% CI 1.1–4.7) were significantly associated with MDR-TB. Similarly, poor treatment outcomes, including defaulters (OR 7.5, 95% CI 1.6–34.7) and treatment failure (OR 85.5, 95% CI 17.6–414.4), as well as chronic alcohol drinking (OR 2.2, 95% CI 1.2–4.1) and the type of TB contact (OR 2.3, 95 CI 1.2–4.3), were significantly associated with MDR-TB (Table 2).

In contrast, HIV/AIDS comorbidity (OR 0.1, 95% CI 0.02–0.3), asthma comorbidity (OR 0.1, 95% CI 0.04 – 0.5), as well as a lack of a formal education (OR 0.5, 95% CI 0.2–1.1) and primary education (OR 0.6, 95% CI 0.3–1.2) were not significant risk factors for MDR-TB (Table 3).

### 3.3. Predictors of MDR-TB

On multivariate analysis, the regression model showed that rural residency (OR (95% CI) = 3.11 (1.2–8.2), a history of smoking (OR (95% CI) = 4 (1.2–13.2), and poor previous TB treatment outcomes, namely, treatment failure (OR (95% CI) = 56.9 (10.2–319.2), were predictors of MDR-TB (Table 4).

## 4. Discussion

We believe that this is the first comprehensive study conducted in Sudan to provide information regarding the predictors of MDR-TB to help establish potential policies to control MDR-TB in the country. History of treatment failure, being a rural resident, and a history of smoking were found to be significantly associated with the occurrence of MDR-TB on multivariate analysis (Table 4).

The findings regarding the higher rates of MDR-TB among rural residents is similar to the findings of a study that was undertaken in East Shoe, Ethiopia [28], and a previous study that was conducted in Sudan where defaulting on TB treatment was associated with rural residency [35]. Furthermore, being a farmer in Ethiopia was also reported to be significantly associated with MDR-TB [30]. Another study that was conducted in Amhara National Regional State, Ethiopia, revealed that a low monthly income was also one of the MDR-TB predictors [31], which may be seen in more rural settings. A history of smoking was also found to be associated with the occurrence of MDR-TB (Table 2), similar to studies that were conducted in Henan province in China, North India, and central Nepal [34,36,37].

Educational status was not significantly associated with MDR-TB in our study. This is similar to two studies that were undertaken in Ethiopia [28,38]. However, this is different from the findings of Rifat et al. [32] and Zhang et al. [34]. We are not sure of the reasons for these differences, and we will be looking at this further. Even though the spread of TB is through direct contact, the current model did not show a significant association between TB contact history and MDR-TB. This finding is in line with a previous study in Iran, where there appeared to be no association between previous TB contact and MDR-TB [39]. However, other studies have found an association between TB contact history and MDR-TB [28,29,31,38,40]. Again, we will be looking at this further.

MDR-TB is most prevalent among patients who have been previously prescribed anti-TB medicines as compared to the incidence of resistant TB among new cases [41]. Our study also revealed a significant association between MDR-TB and failure cases. Mulisa et al. also reported that treatment failure was statistically associated with the development of MDR-TB [30].

Our findings did not show any association with some socio-demographic factors, such as sex, annual income, number of family members, age, and occupation (Table 3); whereas, one study that was undertaken in India reported that females are more prone to MDR-TB than males [42]. In Bangladesh, families of more than four members were significantly associated with MDR-TB [40], differing though from our findings. Jitmuang et al. reported that those under 65 years are more susceptible to MDR-TB [43], which is again in contrast to our findings. Similarly, in Shanghai, patients 46 years old and above was seen three times more likely to get MDR-TB when compared to those less than 46 years old [8]. We are again not sure of the reasons behind these differences and will be exploring this in future studies.

TB is prevalent among HIV/AIDS patients, with the rate of acquisition of TB estimated to be between 16–27 times more prevalent among HIV/AIDS as compared to those without this disease [44,45]. However, our model did not show any significant association with HIV/AIDS on multivariate analysis (Table 4). This is similar to the finding that was reported by Mulu et al., where no significant association was found [31]; however, our finding is different to other studies that have reported an association between HIV/AIDS and the existence of MDR-TB [30,33]. We will also be exploring this further in future studies given the comments from the WHO in their publications [44,45].

A history of drinking alcohol was not an MDR-TB predictor on the multivariate analysis in our study. This is similar to the findings in the Oromia region in Ethiopia, Lithuania, and Botswana [30,46,47]. Similarly, Marahatta et al. reported no evidence of a significant association between alcohol and MDR-TB in Nepal [36]. There was also no association between a history of imprisonment and MDR-TB in our study, which is similar to studies in Ethiopia [28,30]. However, in other studies, prisoners, in general, have been a particular concern, as they tend to have an appreciably higher prevalence of TB than the general population, with prisoners typically being at high risk for developing TB due to issues, such as substance abuse, homelessness, and mental illness [48]. These factors, combined with poor ventilation, overcrowding, and poor diet in prisons, can enhance TB rates [48].

Overall, the differences and similarities of factors that are associated with the development of MDR-TB among regions and countries highlights the importance of conducting local studies to determine local factors that are robustly associated with MDR-TB. However, any appreciable differences in the findings between countries are a cause of concern, and they point to the need for additional research to clarify the situation. Consequently, patients with locally identified risk factors need to be a high priority in any local TB program until the results of any further research are known. In turn, this would help the health authorities to curb the MDR-TB in their country [30].

In summary, on multivariate analysis, living in rural areas, history of smoking, and poor treatment outcomes, including treatment failure, were found to be the predictors of MDR-TB in Sudan. Consequently, the national TB program, which is the responsible body for the control of the disease in Sudan, should maximize efforts to combat TB and anti-TB drug resistance. The National TB program should complete the ongoing national survey to more accurately ascertain the incidence of MDR-TB in Sudan. Besides increasing the number of treatment centers and the free provision of TB medicines, there is also a need for programs to address and overcome the problems that are associated with rural residency. This could include the improvement of transportation to the centers if their costs are a concern. There is also a need to progress policies to reduce smoking, building on the momentum that has been created in Sudan by recent initiatives (https://tobaccoatlas.org/country/sudan/). The successful detection of new cases and their proper management will lead to appreciable improvements in treatment outcomes of patients with TB in Sudan in the future.

## 5. Study Limitations

Case-control was the study design employed. This type of study is prone to recall bias, which is the main drawback. However, this is the most applicable study design in this case. We believe our findings are robust in providing future directions, despite these limitations.

## 6. Conclusions and Recommendations

The present study showed that living in rural areas, smoking, and previous poor treatment outcomes, were significantly associated with the occurrence of MDR-TB. The reinforcement of health education, total DOT programs coverage for all TB patients, and working on identified determinants are highly recommended to reduce the spread of MDR-TB in Sudan. NTP-Sudan should also coordinate with the private sector to refer TB patients to appropriate TB management units (TBMUs) to ensure that the management of TB cases is under program supervision.

In addition, addressing concerns with rural residency and smoking where these occur, together with an implementation of DOT programs and health education, will be crucial in stopping the spread of MDR-TB in Sudan. We will be monitoring such developments in the future.

## Figures and Tables

**Table 1:** Socio-demographic and clinical characteristics of the study participants.

Variable	Case (%)	Control (%)	Total (%) ^a^	*P*-value
**Age group**				
18–39	52 (68.4)	65 (60.7)	117 (63.9)	
40–54	16 (21.1)	24 (22.4)	40 (21.9)	0.40
≥55	8 (10.5)	18 (16.8)	26 (14.2)	
**Sex**				
Male	59 (77.6)	81 (75.7)	140 (76.5)	0.76
Female	17 (22.4)	26 (24.3)	43 (23.5)	
**Occupation**				
Daily laborer	64 (84.2)	85 (81)	149 (82.3)	
House-wife	9 (11.8)	15 (14.3)	24 (13.3)	0.86
Employee	3 (3.9)	5 (4.8)	8 (4.4)	
**Educational status**				
No formal education	14 (18.4)	28 (26.9)	42 (23.3)	
Primary school	32 (42.1)	48(46.2)	80 (44.4)	0.16
Higher secondary school	30 (39.5)	28 (26.9)	58 (32.2)	
and above	
**Residency**				
Rural	55 (72.4)	42 (39.6)	97 (53.3)	<0.05 *
Urban	21 (27.6)	64 (60.4)	85 (46.7)	
**Number of Family members**				
2 or less	3 (3.9)	2 (2)	5 (2.8)	
3–6	28 (36.8)	33 (32.4)	61 (34.3)	0.51
More than 6	45 (59.2)	67 (65.7)	112 (62.9)	
**Annual income (SDG)**				
Less than 60000	44 (75.9)	68 (84)	112 (80.6)	
60000–100000	13 (22.4)	12 (14.8)	25 (18)	0.43
More than 100000	1 (1.7)	1 (1.2)	2 (1.4)	
**HIV infection**				
Positive	2 (2.7)	30 (28.3)	32 (17.9)	<0.05 *
Negative	71 (97.3)	76 (71.7)	147 (82.1)	
**Smoking**				
Yes	45 (59.2)	46 (43)	91 (49.7)	0.03 *
No	31 (40.8)	61 (57)	92 (50.3)	
**Chronic antacid use**				
Yes	16 (21.6)	36 (35)	52 (29.4)	0.06
No	58 (78.4)	67 (65)	125 (70.6)	
**Drug addiction**				
Yes	18 (23.7)	13 (13.1)	31 (17.7)	0.07
No	58 (76.3)	86(86. 9)	144 (82.3)	
**History of TB contact**				
Yes	34 (45.3)	27 (26.7)	61 (34.7)	0.01 *
No	41 (54.7)	74 (73.3)	115 (65.3)	
**Previous TB treatment outcome**				
Defaulter	17 (23.9)	43 (40.2)	60 (33.7)	
Failure	45 (63.4)	10 (9.3)	55 (30.9)	<0.05 *
Completed	7 (9.9)	16 (15)	23 (12.9)	
Cured	2 (2.8)	38 (35.5)	40 (22.5)	
**Diabetes mellitus (DM)**				
Yes	12(15.8)	18(17)	30 (16.5)	0.83
No	64(84.2)	88(83)	152 (83.5)	
**Asthma**				
Yes	3 (3.9)	25 (24)	28 (15.6)	<0.05 *
No	73 (96.1)	79 (76)	152 (84.4)	
**History of imprisonment**				
Yes	22 (28.9)	16 (15.1)	38 (20.9)	0.02 *
No	54 (71.1)	90 (84.9)	144 (79.1)	
**Chronic alcohol drinker**				
Yes	36 (47.4)	31 (29)	67 (36.6)	0.01 *
No	40 (52.6)	76 (71)	116 (63.4)	
**Snuff Chewing**				
Yes	38 (50)	42 (39.3)	80 (43.7)	0.15
No	38 (50)	65 (60.7)	103 (56.3)	

NB: *p*-values were calculated from Chi-square (ꭓ^2^) or Fisher’s exact test; TB = Tuberculosis; *^a^* Not all variables sum up to 183 due to missing data; * statistically significant at *p* < 0.05.

**Table 2:** Association of clinical and behavioral factors with multidrug-resistant tuberculosis (MDR-TB) among MDR-TB cases.

Variables		Cases Number (%) (*n* = 76)	Control Number (%) (*n* = 107)	*p*-Value	Crude OR (95% CI)
HIV infection	Positive	2 (2.7)	30 (28.3)	<0.001	0.1 (0.02–0.3) *
	Negative	71 (97.3)	76 (71.7)		1
Smoking	Yes	45 (59.2)	46 (43)	0.03	1.93 (1.1–3.5) *
	No	31 (40.8)	61 (57)		1
Chronic antacid use	Yes	16 (21.6)	36 (35)	0.06	0.5 (0.3–1.0)
	No	58 (78.4)	67 (65)		1
Drug addiction	Yes	18 (23.7)	13 (13.1)	0.07	2.1 (0.9–4.5)
	No	58 (76.3)	86(86. 9)		1
History of TB contact	Yes	34 (45.3)	27 (26.7)	0.01	2.3 (1.2–4.3) *
	No	41 (54.7)	74 (73.3)		1
	Defaulter	17 (23.9)	43 (40.2)	0.01	7.5 (1.6–34.7) *
Previous TB treatment outcome	Failure	45 (63.4)	10 (9.3)	<0.001	85.5 (17.6–414.4) *
	Completed	7 (9.9)	16 (15)	0.01	8.31 (1.56–44.45) *
	Cured	2 (2.8)	38 (35.5)		1
Diabetes mellitus (DM)	Yes	12 (15.8)	18(17)	0.83	0.92 (0.41–2.04)
	No	64 (84.2)	88 (83)		1
Asthma	Yes	3 (3.9)	25 (24)	0.001	0.13 (0.04–0.45) *
	No	73 (96.1)	79 (76)		1
History of imprisonment	Yes	22 (28.9)	16 (15.1)	0.03	2.29 (1.11–4.74) *
	No	54 (71.1)	90 (84.9)		1
Chronic alcohol drinker	Yes	36 (47.4)	31 (29)	0.01	2.21 (1.19–4.08) *
	No	40 (52.6)	76 (71)		1
Snuff Chewing	Yes	38 (50)	42 (39.3)	0.15	1.55 (0.86–2.80)
	No	38 (50)	65 (60.7)		1

NB: OR = odds ratio; CI = confidence interval; HIV = human immunodeficiency virus; TB = Tuberculosis; MDR-TB = multidrug-resistant tuberculosis; * statistically significant at *p* < 0.05.

**Table 3:** Association of socio-demographic factors with MDR-TB among the presumptive MDR-TB cases.

Variables		MDR-TB Number (%) (*n* = 76)	Non-MDR-TB Number (%) (*n* = 107)	*p*-Value	Crude OR (95% CI)
Age (in years)	18–39	52 (68.4)	65 (60.7)	0.21	1.8 (0.7–6.2)
	40–54	16 (21.1)	24 (22.4)	0.45	1.5 (0.5–4.4)
	≥55	8 (10.5)	18 (16.8)		1
Sex	Male	59 (77.6)	81 (75.7)	0.76	0.9 (0.5–1.8)
	Female	17 (22.4)	26 (24.3)		1
Occupation	Daily laborer	64 (84.2)	85 (81)	0.76	1.3(0.3–5.5)
	House-wife	9 (11.8)	15 (14.3)	>0.05	1.0 (0.2–5.2)
	Employee	3 (3.9)	5 (4.8)		1
Educational status	No formal education	14 (18.4)	28 (26.9)	0.07	0.5 (0.2–1.1)
	Primary school	32 (42.1)	48(46.2)	0.17	0.6 (0.3–1.2)
	Higher secondary school	30 (39.5)	28 (26.9)	0.08	1
	and above				
Residency	Rural	55 (72.4)	42 (39.6)	<0.01	4.0 (2.1–7.5) *
	Urban	21 (27.6)	64 (60.4)		1
Family members (n)	2 or less	3 (3.9)	2 (2)		1
	3–6	28 (36.8)	33 (32.4)	0.55	0.6 (0.1–3.6)
	More than 6	45 (59.2)	67 (65.7)	0.39	0.5 (0.07–2.8)
	Less than 60000 SDG	44 (75.9)	68 (84)	0.76	0.7 (0.04–10.6)
Annual income	60000–100000 SDG	13 (22.4)	12 (14.8)	0.96	1.1 (0.1–19.3)
	More than 100000 SDG	1 (1.7)	1 (1.2)		1

NB: MDR-TB = multidrug-resistant tuberculosis; OR = odds ratio; CI = confidence interval * statistically significant at *p* < 0.05.

**Table 4:** Predictors of MDR-TB among resistant cases.

Variables		Cases Number (%) (*n* = 76)	Control Number (%) (*n* = 107)	Crude OR (95%CI)	Adjusted OR (95% CI)
Residency	Rural	55 (72.4)	42 (39.6)	4 (2.10–7.5) *	3.11 (1.2–8.2) *
	Urban	21 (27.6)	64 (60.4)	1	1
Smoking	Yes	45 (59.2)	46 (43)	1.9 (1.1–3.5) *	4 (1.2–13.2) *
	No	31 (40.8)	61 (57)	1	1
HIV infection	Positive	2 (2.7)	30 (28.3)	0.1 (0.02–0.3) *	0.1 (0.02–1.1)
	Negative	71 (97.3)	76 (71.7)	1	1
History of TB contact	Yes	41 (54.7)	74 (73.3)	2.3 (1.2–4.3) *	2.3 (0.8–6.5)
	No	41 (54.7)	54 (72)	1	1
	Defaulter	17 (23.9)	43 (40.2)	7.5 (1.6–34.7) *	3 (0.6–15.8)
Previous TB treatment outcome	Failure	45 (63.4)	10 (9.3)	85.5 (17.6–414.4) *	56.9 (10.2–319.2) *
	Completed	7 (9.9)	16 (15)	8.3 (1.6–44.5) *	4.1 (0.7–24.5)
	Cured	2 (2.8)	38 (35.5)	1	1
Asthma	Yes	3 (3.9)	25 (24)	0.1 (0.04–0.5) *	0.3 (0.04–1.8)
	No	73 (96.1)	79 (76)	1	1
History of imprisonment	Yes	22 (28.9)	16 (15.1)	2.3 (1.1–4.7) *	1 (0.3–3)
	No	54 (71.1)	90 (84.9)	1	1
Chronic alcohol drinker	Yes	36 (47.4)	31 (29)	2.2 (1.2–4.1) *	1.1 (0.3–3.4)
	No	40 (52.6)	76 (71)	1	1

NB: OR = odds ratio; CI = confidence interval; HIV = human immunodeficiency viruses; TB = Tuberculosis; * statistically significant at *p* < 0.05.
